# The anti-cholestatic effects of *Coptis chinensis* Franch. alone and combined with *Tetradium ruticarpum* (A. Jussieu) T. G. Hartley: dual effects on fecal metabolism and microbial diversity

**DOI:** 10.3389/fphar.2024.1372527

**Published:** 2024-03-08

**Authors:** Jun Han, Peijie Wu, Zongying Xu, Chao Liu, Qian Chen, Fenghua Zhang, Huan Tao, Dan Luo, Li Zhou, Bo Wang, Zhe Gao, Tao Shen, Yueqiang Wen, Han Yu

**Affiliations:** ^1^ School of Basic Medicine, Chengdu University of Traditional Chinese Medicine, Chengdu, China; ^2^ School of Basic Medical Sciences, Guangzhou University of Chinese Medicine, Guangzhou, China; ^3^ Hospital of Chengdu University of Traditional Chinese Medicine, Chengdu, China; ^4^ Cangxi Traditional Chinese Medicine Hospital, Guangyuan, China; ^5^ School of Medicine and Life Sciences, Chengdu University of Traditional Chinese Medicine, Chengdu, China

**Keywords:** anti-cholestatic effects, *Coptis chinensis* Franch., *Tetradium ruticarpum* (A. Jussieu) T. G. Hartley, fecal metabolism, fecal microbial diversity

## Abstract

**Introduction:** Drug dosages and combinations are the main factors that affect the efficacy of pleiotropic traditional Chinese medicine (TCM). *Coptis chinensis* Franch. (CF) is a representative TCM with multiple effects and is often combined with *Tetradium ruticarpum* (A. Jussieu) T. G. Hartley (TR) to treat cholestasis. The present study assessed the influence of CF dose and its combination with TR on the efficacy of CF in cholestasis treatment, including their effects on fecal metabolism and fecal microorganisms.

**Methods:** Rats with α-naphthylisothiocyanate (ANIT, 50 mg/kg)-induced cholestasis were administered low (0.3 g/kg) and high (0.6 g/kg) doses of CF, as well as CF combined with TR at doses of 0.6 g/kg and 0.9 g/kg, respectively. The anti-cholestatic effects of these treatments were assessed by determining their anti-inflammatory, hypolipidemic, and anti-oxidative stress properties. Additionally, fecal metabolomics and fecal microorganisms were analyzed.

**Results:** Low dose CF had a more potent hypolipidemic effect than high dose CF, whereas high dose CF had more potent anti-inflammatory and anti-oxidative stress effects. Combination with TR enhanced the hypolipidemic effect, but antagonized the anti-inflammatory effect, of CF. Analyses of fecal metabolomics and fecal microorganisms showed differences in the regulation of lipid- and amino acid metabolism-related pathways, including pathways of linoleic acid, tyrosine, and arachidonic acid metabolism, and amino acid biosynthesis between different doses of CF as well as between different doses of CF in combination with TR. These differences may contribute to differences in the anti-cholestatic effects of these preparations.

**Conclusion:** CF dose influences its anti-cholestatic efficacy. The combination with TR had synergistic or antagonistic effects on the properties of CF, perhaps by altering fecal metabolism and fecal microbial homeostasis.

## 1 Introduction

The clinical application of Chinese botanical drugs has been found to play a vital role in maintaining human health. *Coptis chinensis* Franch. (CF), one of the most commonly used botanical drugs in traditional Chinese medicine (TCM), is widely used in the treatment of various diseases, such as coronary heart disease, cholestasis, and gastroenteritis (Wang et al., 2019). The *"Pharmacopoeia of the People’s Republic of China,”* an official revision of the Chinese pharmacopeia, has summarized its efficacy in three main areas: clearing heat, eliminating dampness, and detoxification, regarding CF as crucial in the treatment of cholestasis (Commission, 2020), a common disease characterized by abnormal bile secretion or excretion (Song et al., 2022).

The effects of CF in TCM may depend on its dosage and its combination with other botanical drugs (Deng, 2018). For example, the *Huanglian Jiedu decoction*, a combination of CF with *Cortex phellodendri chinensis*, *Radix scutellariae*, and *Fructus gardeniae* in a 3:2:2:3 ratio, showed antipyretic effects by disrupting the JAK2/STAT3 and MAPK signaling pathways (Lu et al., 2020a; Li et al., 2021). Similarly, the *Zhuyu pill*, a combination of CF with *Tetradium ruticarpum* (A. Jussieu) T. G. Hartley (TR) in a 1:1 ratio, exhibited a significant hypolipidemic effect through the gut-liver axis and the NF-κB signaling pathway (Pan et al., 2023; Xu et al., 2023). Although CF has shown therapeutic efficacy in various diseases, further investigations are required to explore the impact of different dosages of CF or combinations with other agents on its effectiveness. Therefore, in treating cholestasis, the CF dose and its possible combination with other botanical drugs may vary according to specific conditions.

The efficacy of TCM botanical drugs in clearing heat, eliminating dampness, and detoxification is frequently assessed by measuring its anti-inflammatory, hypolipidemic, and anti-oxidative stress effects, respectively (Zhang et al., 2018; Lu et al., 2020b; Shou and Shaw, 2023). Because mechanisms involved in the pathology of cholestasis include inflammatory reactions, lipid metabolism disorders, and increased oxidative stress (Li et al., 2022a; Labiano et al., 2022), CF may be effective in treating cholestasis by having anti-inflammatory effects, promoting lipid metabolism, and reducing oxidative stress. Our previous study (Han et al., 2023a) found that CF and TR in a 1:1 ratio could treat cholestasis by regulating lipid and bile acid metabolism. It was unclear, however, whether the effectiveness of CF in cholestasis was due to its anti-inflammatory effects, its enhancement of lipid metabolism, or its resistance to oxidative stress. More, the therapeutic efficacy of CF in the treatment of cholestasis may be dose dependent or may be altered when combined with TR.

The effects of CF doses, alone or combined with TR, were therefore evaluated in a rat model of cholestasis. In addition, the biological mechanisms leading to differences in efficacy were analyzed by fecal metabolome sequencing and fecal microbial homeostasis. The effects of CF dosage and compatibility on its therapeutic efficacy in the treatment of cholestasis were analyzed, providing a biological basis for the effects of dosage and compatibility on the efficacy of drugs in TCM.

## 2 Materials and methods

### 2.1 Reagent preparation

*Coptis chinensis* Franch. and *T. ruticarpum* (A. Jussieu) T. G. Hartley were purchased from Beijing Tongrentang Co., Ltd. (China). Ursodeoxycholic acid (UDCA), α-naphthylisothiocyanate (ANIT), and sodium pentobarbital were obtained from Merck Pharmaceuticals, Inc. (Germany); and olive oil was from Shanghai Yi En Chemical Technology Co.

### 2.2 Drug preparation and identification of active metabolites

The characteristics of the botanical drugs used in these four combinations are shown in Table 1. The dosage of each medicinal plant was based on the dosage range specified in the *"Pharmacopoeia of the People’s Republic of China.”* Each group of botanical drugs was prepared according to the decoction method stipulated in the "14th Five-Year Plan” of the National Higher Education Materials on TCM. Briefly, each group of botanical drugs was mixed separately and immersed in 20 times the volume of purified water. Heating was started after 30 min and continued for an additional 30 min. The liquid was filtered and collected; the above procedure was repeated; and the twice-collected liquid was mixed and stored at −20°C.

**TABLE 1:** Characteristics of the botanical drugs in this study.

Group	Chinese name	Botanical name^a^	Genus family	Batch number	Medicinal parts	Origin	Weight (g)
CF_L	Huanglian	*Coptis chinensis* Franch	*Ranunculaceae*	220701	Dried root	Chongqing, China	3
CF_H	Huanglian	*Coptis chinensis* Franch	*Ranunculaceae*	220701	Dried root	Chongqing, China	6
CT_L	Huanglian	*Coptis chinensis* Franch	*Ranunculaceae*	220701	Dried root	Chongqing, China	3
	Wuzhuyu	*Tetradium ruticarpum* (A. Jussieu) T. G. Hartley	*Rutaceae*	220416008	Dried and ripe seed	Guizhou, China	3
CT_H	Huanglian	*Coptis chinensis* Franch	*Ranunculaceae*	220701	Dried root	Chongqing, China	6
	Wuzhuyu	*Tetradium ruticarpum* (A. Jussieu) T. G. Hartley	*Rutaceae*	220416008	Dried and ripe seed	Guizhou, China	3

^a^ The plant name was verified using http://www.theplantlist.org.

The active metabolites in these herbal decoctions were identified by Q-Orbitrap high-resolution LC-MS. Information on the equipment used, sample handling procedures, mass spectrometry conditions, and chromatographic conditions is included in Supplementary Material 1. The decoctions were subjected to high-resolution liquid chromatography, with the results collected using CD 3.3 (Compound Discoverer 3.3, Thermo Fisher Scientific) and compared with the database (mzCloud, https://www.mzcloud.org/) to identify the compounds. Determination of the activity of orally administered Chinese botanical drugs requires overcoming barriers due to absorption, distribution, metabolism, and excretion processes. The active metabolites obtained from the comparison were further screened by assessing their oral bioavailability (OB) (Xu et al., 2012) and drug-likeness (DL), with substances having a DL index ≥0.18 considered highly drug-like (Guo et al., 2019). The OB and DL of each active metabolite was assessed using the TCMSP database (https://old.tcmsp-e.com/molecule.php?qn=2649). Active metabolites were screened based on their OB, DL, and comparison with the database (mzCloud, https://www.mzcloud.org/), with active metabolites regarded as molecules with an OB ≥ 30%, a DL ≥ 0.18, and score of an mzCloud best match ≥80.

### 2.3 Animals and treatments

Thie animal protocol was approved by the Animal Ethics Committee of Chengdu University of Traditional Chinese Medicine (Experimental Animal Welfare Ethics Review Certificate No. 2023024), and the animals were handled strictly according to internationally recognized animal management and rules.

**FIGURE 1:**
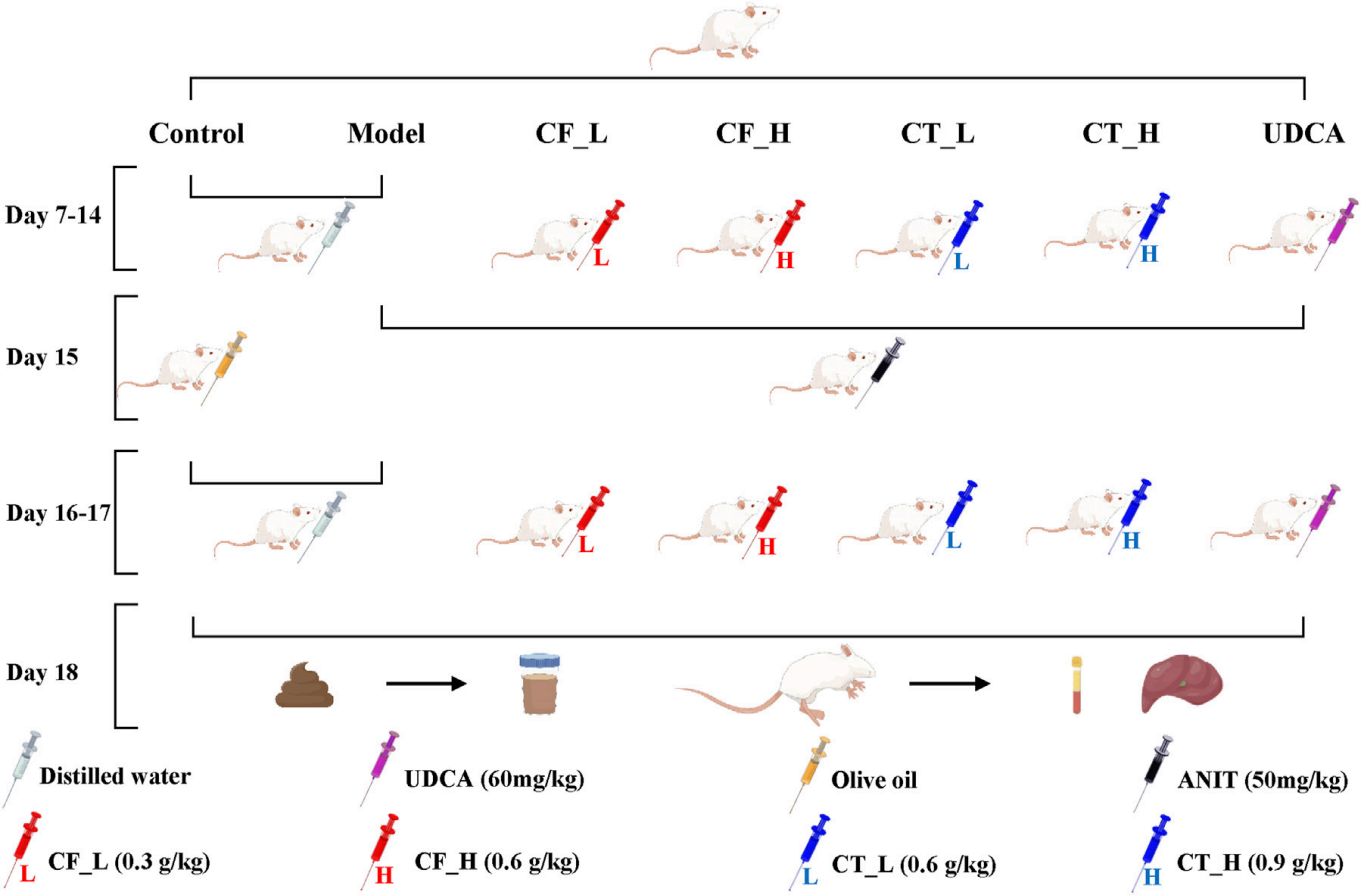
Animal Experimental Procedures. Cholestasis model construction; gavage administration; fecal collection; anesthesia; blood and liver tissue collection.

The rat model, the administration of drugs by gavage, and the procedures for anesthesia, blood collection, and extraction of liver tissues have been described (Yu et al., 2022a). Briefly, 42 healthy male Sprague Dawley rats weighing 250 ± 20 g were purchased from Chengdu Da Shuo Experimental Animal Co., Ltd., production license number SCXK (CHUAN) 2020-0030. After 6 days of acclimatization feeding, the rats were randomly divided into seven groups of six rats each: the control group (Control), the model group (Model), the CF low dose group (CF_L), the CF high dose group (CF_H), the CF low dose CF plus TR group (CT_L), the CF high dose plus TR group (CT_H), and the positive control group (UDCA). Figure 1. shows the entire process of model construction and drug administration by gavage. Cholestasis in rats was induced by intragastric administration of ANIT, at a dose of 50 mg/kg, dissolved in olive oil. Intragastric administration of CF and CT was determined relative to body surface area. After gavage administration on day 17, all rats were fasted for 16 h. After collecting fresh feces from each rat on day 18, the rats were anesthetized by intraperitoneal injection of sodium pentobarbital 30 mg/kg. Blood samples were collected, the rats were sacrificed, and liver tissue samples were collected. Feces and liver tissues were frozen and stored at −80°C.

### 2.4 Liver function assays

Rat blood samples were centrifuged at 3,500 × g for 15 min at 4°C to obtain serum. ALT, AST, ALP, γ-GT, DBIL, TBIL, and TBA concentrations in serum were determined using a fully automated biochemical analyzer (BS-240VET). Liver tissue samples were fixed with 4% paraformaldehyde, washed with water, dehydrated, embedded, and sectioned. The sections were stained with HE and examined under a microscope for pathological determination.

### 2.5 Assessments of anti-inflammatory, hypolipidemic, and anti-oxidative stress effects

The anti-inflammatory effects of treatment were evaluated by determining the levels of expression of TNF-α, IL-1β, IL-6, and IL-10 mRNAs in rat liver tissue by real-time quantitative PCR (qRT-PCR), using qRT-PCR primers (Table 2) based on the mRNA sequences in the NCBI database.

**TABLE 2:** qRT-PCR primers used in this study.

Genes	Forward primer (5′–3′)	Reverse primer (5′–3′)	Product length (bp)
TNF-α	GGT​GCC​TAT​GTC​TCA​GCC​TCT​T	GCC​ATA​GAA​CTG​ATG​AGA​GGG​AG	138
IL-1β	GTG​GCT​GTG​GAG​AAG​CTG​TGG	CGG​AGC​CTG​TAG​TGC​AGT​TGT​C	147
IL-6	CCA​CTC​CCA​ACA​GAC​CTG​TCT​A	CTG​CAA​GCC​AGT​TTG​GTA​GCA​TC	192
IL-10	AGC​CTT​ATC​GGA​AAT​GAT​CCA​G	GGC​CTT​GTA​GAC​ACC​TTG​GT	229

The effect of regulating lipid metabolism was evaluated by staining liver tissue with oil red O, as well as by measuring TC and TG concentrations in blood samples. The serum concentrations of GSH, ROS, NO, MDA, and SOD were determined by micro-enzyme assay (cat. no. A006-2), enzyme-linked immunosorbent assay (cat. no. YJ206302), colorimetry (cat. no. E-BC-K035-S), tribarbituric acid assay (cat. no. A003-1), and hydroxylamine assay (cat. no. A001-1), respectively.

### 2.6 Fecal metabolomics and analysis of microbial diversity

Metabolites in each group of rats were analyzed by gas chromatography-mass spectrometry (GC-MS). The analytical process included metabolite extraction and derivatization, detection by GC-MS, data pre-processing, and statistical analysis. Additionally, 16S rRNA amplicons were sequenced to determine the fecal microbial characteristics of rats in each group. This analysis included DNA extraction, PCR amplification, library construction and sequencing, and bioinformatics analysis. Both tests were performed by Shanghai OE Biomedical Technology Co., Ltd., and their methodology and procedure have been described (Yu et al., 2021). Further details can be found in Supplementary Material 2.

### 2.7 Statistical analysis

Differences among multiple groups were assessed by one-way ANOVA or the Kruskal Wallis test. All statistical analyses were performed using GraphPad Prism 9, with *p* < 0.05 considered statistically significant.

## 3 Results

### 3.1 Active metabolites of drugs in each group

**FIGURE 2:**
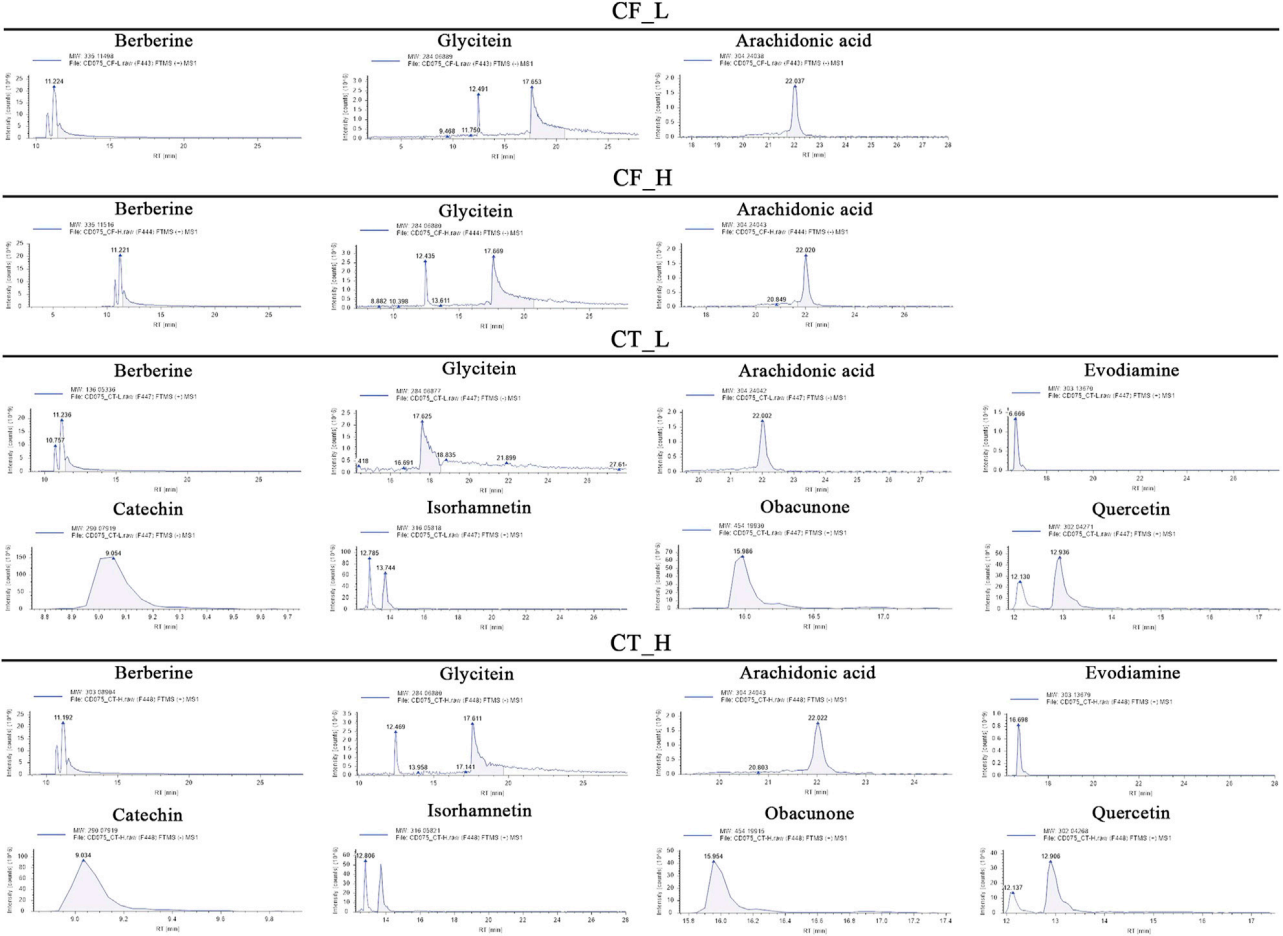
Potential active metabolites of each group for botanical drugs. RT: Chromatographic retention time.

The active metabolites of medicinal plants observed in each group are shown in Figure 2. The primary active metabolites in the CF_L and CF_H groups were berberine, glycitein, and arachidonic acid, whereas the primary active metabolites in the CT_L and CT_H groups were these three metabolites, along with evodiamine, catechin, isorhamnetin, obacunone, and quercetin (Table 3).

**TABLE 3:** All active metabolites of the botanical drugs in each group.

Name	Molecular formula	RT (min)	Score of mzCloud best match	OB (%)	DL	Structural formula
Berberine	C_20_H_17_NO_4_	10.78	93.3	36.86	0.78	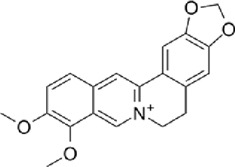
Glycitein	C_16_H_12_O_5_	17.67	97	50.48	0.24	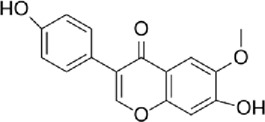
Arachidonic acid	C_20_H_32_O_2_	22.03	91.6	45.57	0.20	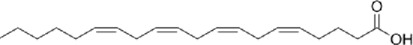
Evodiamine	C_19_H_17_N_3_O	16.71	98	86.02	0.64	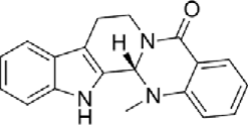
Catechin	C_15_H_14_O_6_	9.04	97	54.83	0.24	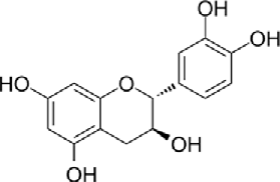
Isorhamnetin	C_16_H_12_O_7_	13.72	99.7	49.60	0.31	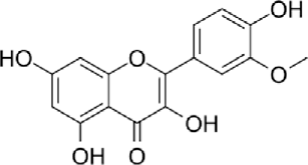
Obacunone	C_26_ H_30_O_7_	15.97	85.6	43.29	0.77	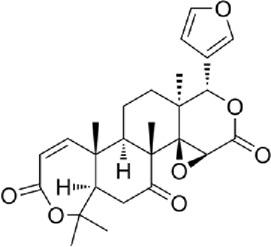
Quercetin	C_15_H_10_O_7_	12.92	95.9	46.43	0.28	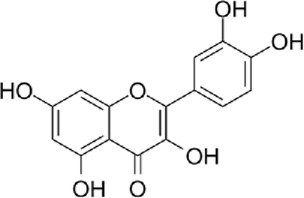

Chemical structural formulas derived from MedChemExpress (https://www.medchemexpress.cn/).

### 3.2 Serum biochemistry and histopathological analysis

**FIGURE 3:**
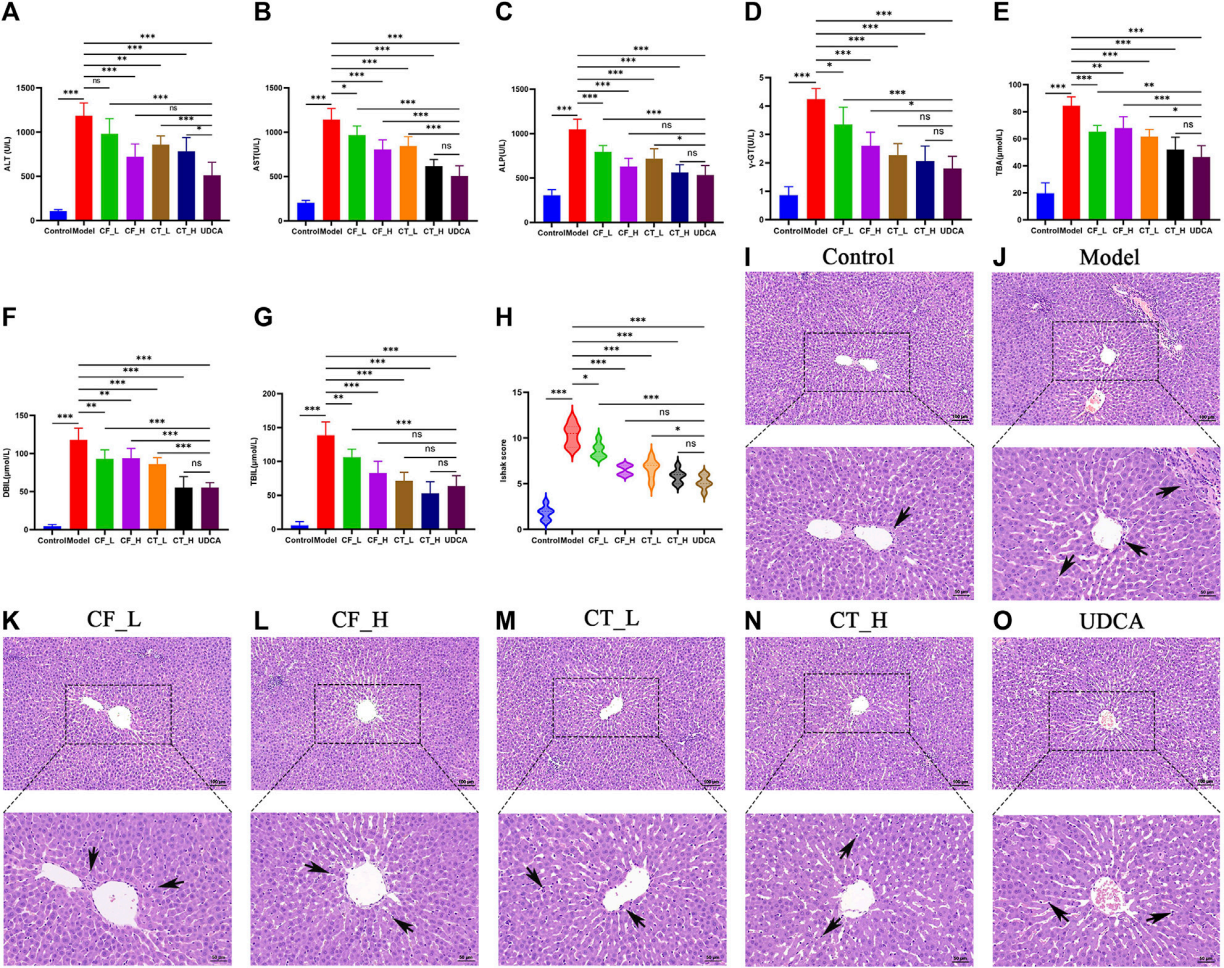
Liver function assay. Serum biochemical indices **(A)** ALT, **(B)** AST, **(C)** ALP, **(D)** γ-GT, **(E)** DBIL, **(F)** TBIL, **(G)** (TBA), **(H)** Liver inflammation and necrosis score, *n* = 6, ***/**/*, *p* < 0.001/*p* < 0.01/*p* < 0.05, ns, not significant. **(I–O)** Histopathological examination at 100x and 200x.

Low and high doses of CF, as well as their combinations with TR, exhibited significant anti-cholestatic activity, with a therapeutic efficacy similar to that of UDCA (Figures 3A–G). Pathohistological examination showed that all groups of botanical drugs reduced the inflammation and liver cell necrosis induced by ANIT (Figures 3H–O). Taken together, these findings indicated that CF and CT had significant therapeutic efficacy against cholestasis.

### 3.3 Assessment of anti-inflammatory effects

**FIGURE 4:**
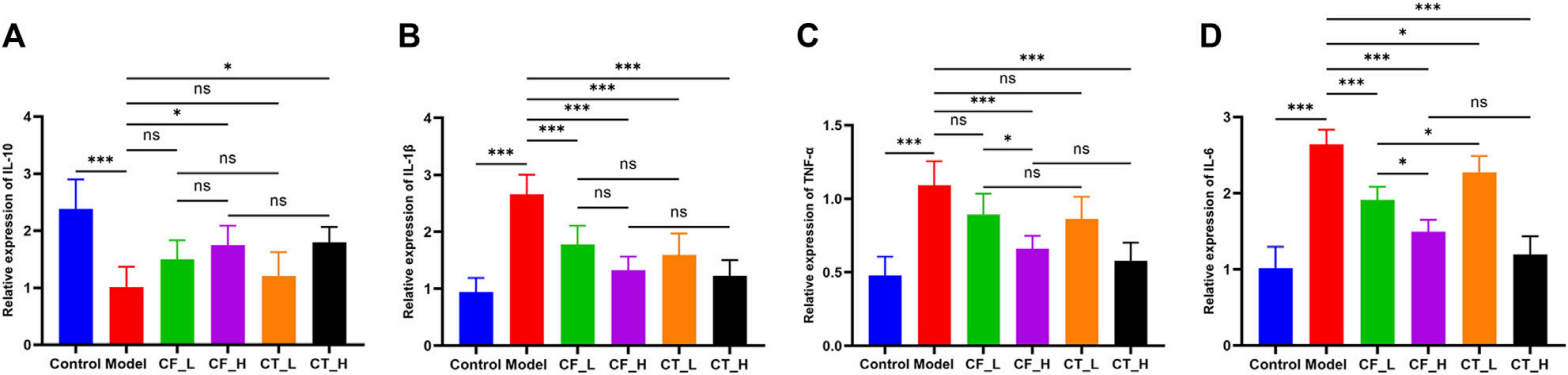
Levels of inflammatory factors in liver tissue of rats in each group. **(A)** IL-10, **(B)** IL-1β, **(C)** TNF-α, **(D)** IL-6. ***/**/*, *p* < 0.001/*p* < 0.01/*p* < 0.05, ns, not significant.

CF_L significantly reduced the concentrations of inflammatory factors IL-1β and IL-6 in liver tissues of cholestatic rats, showing some anti-inflammatory efficacy, whereas CF_H significantly reduced IL-1β, TNF-α, IL-6, and increased IL-10 (Figure 4). CF had significant anti-inflammatory effects, which were more pronounced at high than low CF doses, indicating a quantitative relationship between CF dose and anti-inflammatory activity. The addition of TR, however, did not enhance the anti-inflammatory effects of both low and high doses of CF. Interestingly, CT_H was less effective than CF_L in reducing IL-6 and increasing IL-10 concentrations, suggesting an antagonism between the anti-inflammatory effects of the two drugs in the treatment of cholestasis.

### 3.4 Assessment of hypolipidemic effects

**FIGURE 5:**
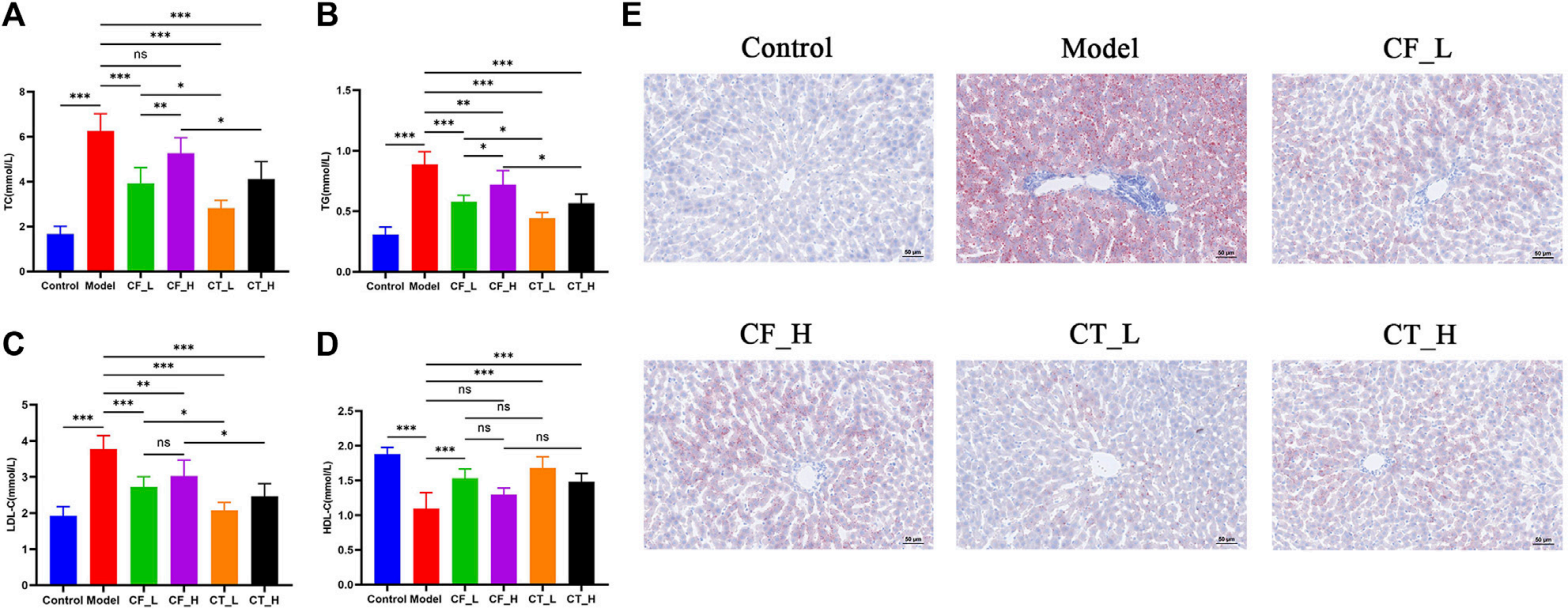
Assessment of lipid metabolism disorders. **(A)** TC, **(B)** TG, **(C)** LDL-C, **(D)** HDL-C, **(E)** Liver tissue oil red O staining. ***/**/*, *p* < 0.001/*p* < 0.01/*p* < 0.05, ns, not significant.

The effects of treatment on lipid metabolism were determined by measuring the levels of lipids in serum and liver samples. Using an automatic biochemical analyzer, lipid-related indices (Figures 5A–D) were detected in the serum of each group of rats. Steatosis was assessed by oil red O staining of liver tissue (Figure 5E). The results indicated that either CF or CT could significantly reduce the accumulation of lipid droplets in the liver tissue of cholestatic rats and improve lipid metabolism disorder. CT_L had a significantly more substantial hypolipidemic effect than CF_L, suggesting that CF and TR had a synergistic hypolipidemic effect in the treatment of cholestasis.

### 3.5 Assessment of anti-oxidative stress effects

**FIGURE 6:**
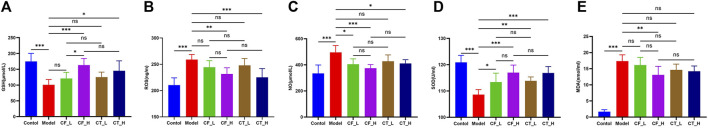
Oxidative stress indicator analysis. **(A)** GSH, **(B)** ROS, **(C)** NO, **(D)** SOD, **(E)** MDA. ***/**/*, *p* < 0.001/*p* < 0.01/*p* < 0.05, ns, not significant.

The anti-oxidative stress effects of treatment were determined by measuring serum concentrations of GSH, ROS, SOD, NO, and MDA in these rats (Figure 6). CF_L did not show a significant anti-oxidative stress effect, whereas CF_H had a highly significant anti-oxidant effect, perhaps because only high doses of CF have anti-oxidant stress efficacy. The addition of TR, however, did not significantly enhance the anti-oxidative stress effect of CF.

### 3.6 Multivariate statistical analysis of GC-MS results

**FIGURE 7:**
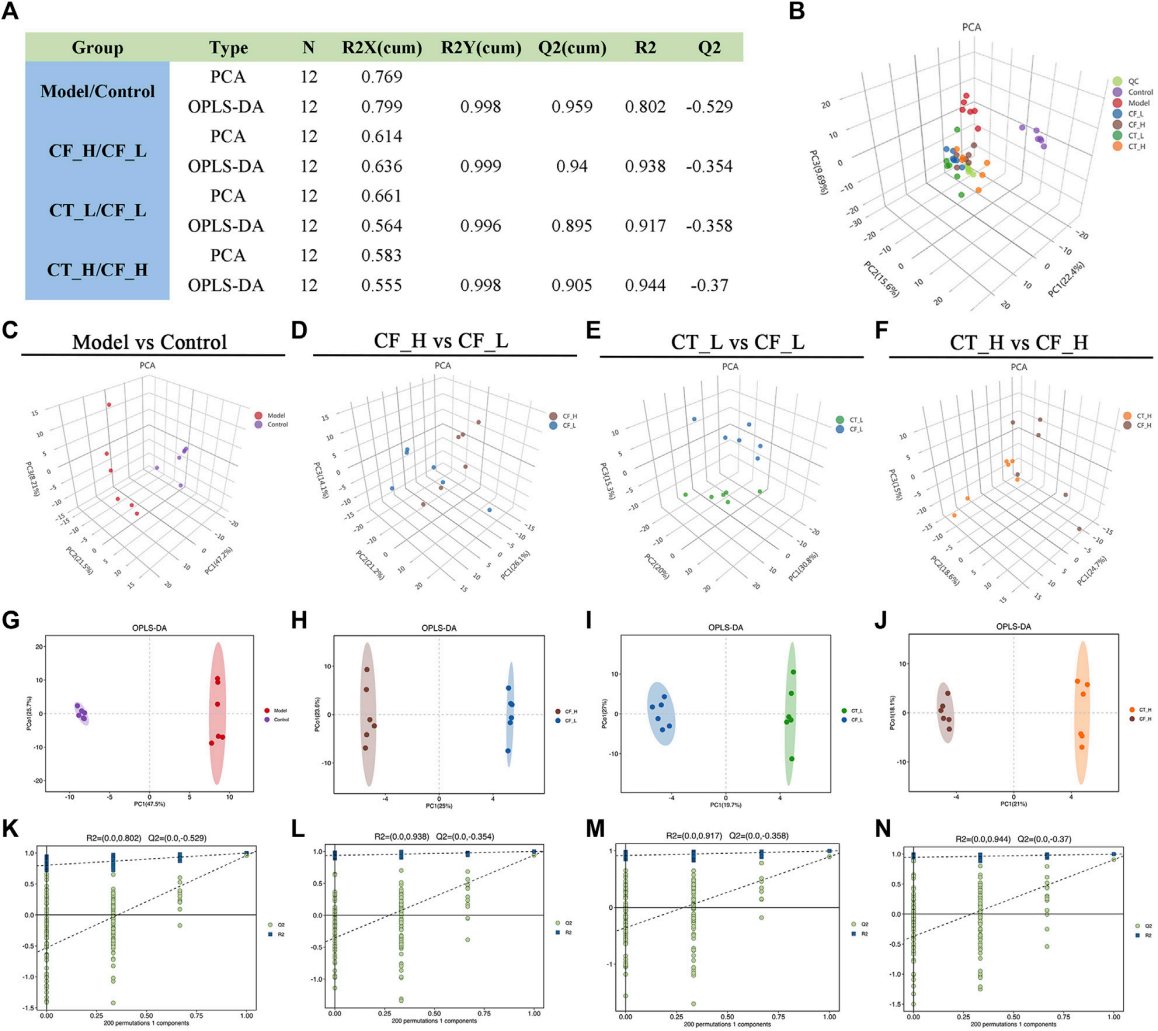
Multivariate statistical analysis of metabolic profiles. **(A)** Parameters of PCA and OPLS-DA, **(B)** PCA score plot of all samples, **(C–F)** PCA analysis, **(G–J)** OLS-DA analysis, **(K–N)** 200-permutation test.

The overall distribution among the samples and the stability of the whole analysis process were initially determined by unsupervised principal component analysis (PCA) (Figures 7B–F). Subsequently, supervised orthogonal partial least squares analysis (OPLS-DA) was used to differentiate the overall differences in metabolic profiles among the groups and to identify the differentially expressed metabolites in these groups (Figures 7G–J). The quality of the model was evaluated by using seven cycles of interactive validation and 200 response ordering tests to prevent model overfitting (Figures 7K–N), with the model parameters shown in Figure 7A. The above analyses showed significant metabolic differences between the CF_H and CF_L, the CT_L and CF_L, and the CT_H and CF_H groups.

### 3.7 Screening of differentially expressed metabolites and pathway enrichment analysis

**FIGURE 8:**
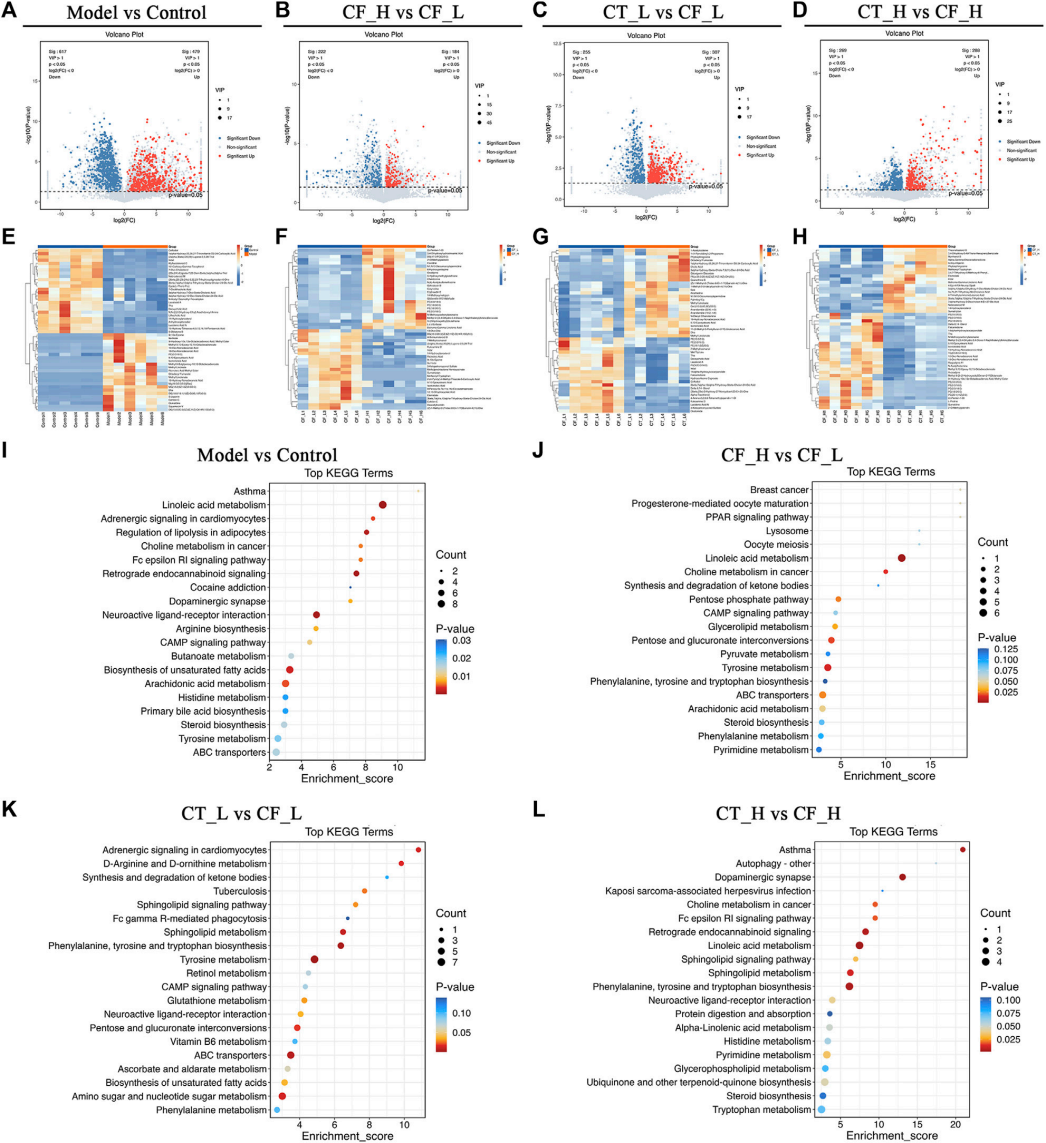
Differential metabolites screening and functional enrichment analysis. **(A–D)** Volcano plot of rat fecal metabolites, **(E–H)** Clustering heat map of rat fecal metabolites, **(I–L)** Differential metabolites KEGG enrichment analysis.

Metabolites differentially expressed by pairs of groups were screened by unidimensional and multidimensional analyses. In the OPLS-DA analysis, variables important in projection (VIP) were used to measure the strength of the influence of the metabolite expression pattern on the classification discrimination of each group of samples and to screen for significant differences in metabolites. Significant differences in the levels of different metabolites between groups were verified by t-tests. The criteria for screening were VIP >1 and *p* < 0.05, with volcano plots (Figures 8A–D) showing the expression trends and numbers of differential metabolites in between group comparisons. Comparisons of the Model and Control groups showed that 617 metabolites were downregulated and 479 upregulated in the former. Comparisons of the CF_H and CF_L groups identified 222 metabolites that were downregulated and 184 that were upregulated in the former. Furthermore, comparisons of the CT_L and CF_L groups found 255 metabolites downregulated and 307 upregulated in the former, whereas comparisons of the CT_H and CF_H groups identified 269 metabolites downregulated and 288 upregulated in the former. Cluster statistical analyses of between group comparisons screened the top 50 differentially expressed metabolites (Figures 8E–H). Differences in the therapeutic effects on cholestasis produced by different doses of CF and by combination with TR may be associated with the differential expression of these metabolites.

The presence of differentially expressed metabolites was likely due to differences in the anti-cholestatic efficacy of various doses of CF and their combinations with TR. Pathway enrichment analysis of these differentially expressed metabolites may reveal the mechanisms underlying these differences in anti-cholestatic metabolic pathways. KEGG enrichment analysis identified overlapping pathways in comparisons of the Model and Control groups (Figure 8I) and the CF_H and CF_L groups. These pathways included those involving ABC transporters, steroid biosynthesis, and linoleic acid, arachidonic acid, and tyrosine metabolism (Figure 8J). These pathways may be responsible for the differences in the anti-inflammatory, hypolipidemic, and anti-oxidative stress effects of high and low doses of CF. Differences in the efficacy of CT_L and CF_L in treating cholestasis were found to be related to differences in the biosynthesis of unsaturated fatty acids, in ABC transporters and in CAMP signaling pathway. The difference in efficacy of CT_H and CF_H was associated with the modulation of linoleic acid metabolism (Figures 8K, L).

### 3.8 Analysis of the diversity of fecal microorganisms

**FIGURE 9:**
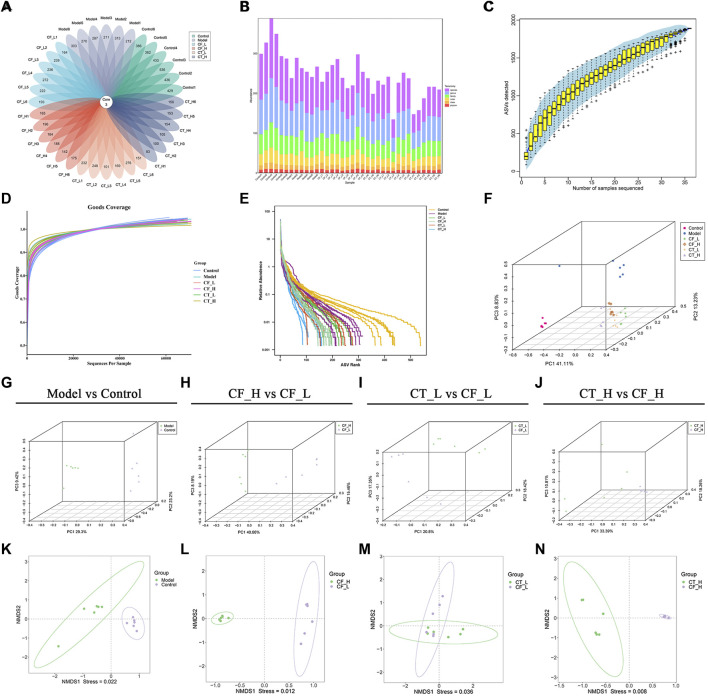
Analysis of fecal microbial diversity. **(A)** Amplicon Sequence Variant (ASVs)-based petal maps. **(B)** Statistics on the distribution of microbial communities in the samples. **(C)** Specaccum Species accumulation curve. **(D)** ASV diversity index dilution curve. **(E)** Rank abundance curve. **(F–J)** PCoA analysis. **(K–N)** NMDS analysis.

Based on the results of amplicon sequence variant (ASV) clustering analysis, the number of shared and unique ASVs in the rat groups were analyzed and plotted as a flower plot (Figure 9A). Three ASVs were shared by the Control, Model, CF_L, CF_H, CT_L and CT_H groups. Figure 9B summarizes the relative abundances of each sample at different taxonomic levels, including of phylum, class, order, family, genus, and species.

The reasonableness of the sequencing volume of the samples was evaluated by alpha diversity analysis, with this evaluation including the adequacy of the sampling volume, and the richness and homogeneity of the species in each sample (Figures 9C–E). Between-group differences in diversity were evaluated by beta diversity analysis, including PCoA and NMDS analyses (Figures 9F–N). Fecal microbial communities were found to differ significantly in the CF_H and CF_L, CT_L and CF_L, and CT_H and CF_H groups. These effects on the composition of the gut microbial community and its function may also be responsible for the differences in the efficacy of CF in treating cholestasis at different doses and after combination with TR.

### 3.9 Multivariate statistical analysis of fecal microorganisms

**FIGURE 10:**
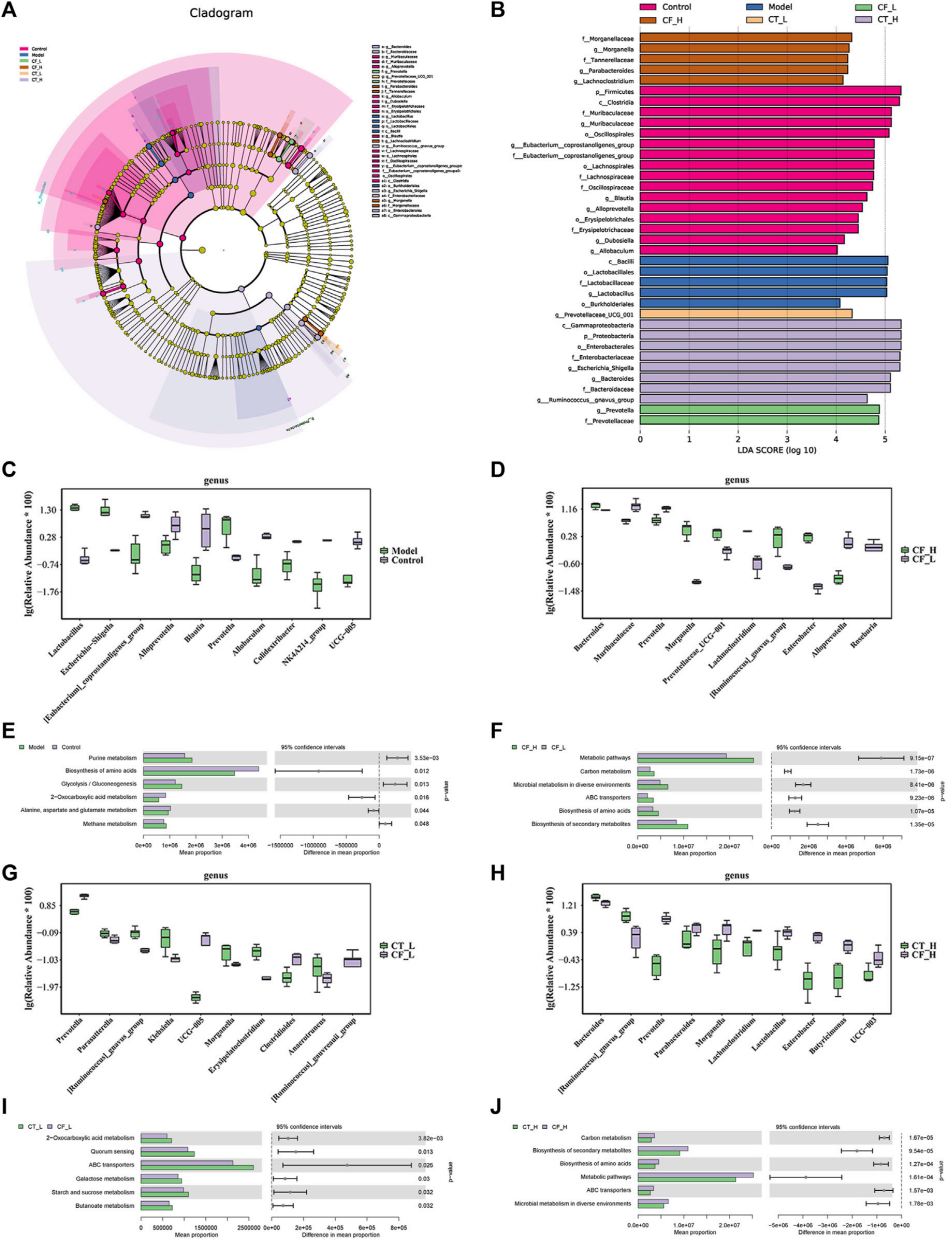
Statistical analysis of microbial multivariate variables. **(A)** LEfSe analysis, differential species annotation branch. **(B)** Histogram of LDA value distribution. **(C, D, G, H)** Top10 species with differential abundance at genus level. **(E, F, I, J)** KEGG function prediction based on 16S rRNA gene sequence.

The species of differentially present microorganisms and their functions were further clarified by LEfSe and PICRUSt2 analyses. Figure 10A shows the significant species with relatively high abundance at the phylum, class, order, family, and genus levels, as determined by LEfSe analyses. Figures 10B–J show the top 10 species that differed at the genus level in between-group comparisons. Comparisons of the microbiomes obtained from the Model and Control groups identified *Alloprevotella* and *Prevotella* as being differentially expressed in the CF_H and CF_L groups, *Prevotella* and *UCG-005* as being differentially expressed in the CT_L and CF_L groups, and *Lactobacillus* and *Prevotella* being differentially expressed in the CT_H and CT_L groups. 16S-based prediction of KEGG function revealed that, in treating cholestasis, the difference between CF_H and CF_L may be related to the biosynthesis of amino acids, the difference between CT_L and CF_L may be related to 2-oxocarboxylic acid metabolism, and the difference between CT_H and CF_H may be related to the biosynthesis of amino acids.

### 3.10 Correlations between metabolites and microorganisms

**FIGURE 11:**
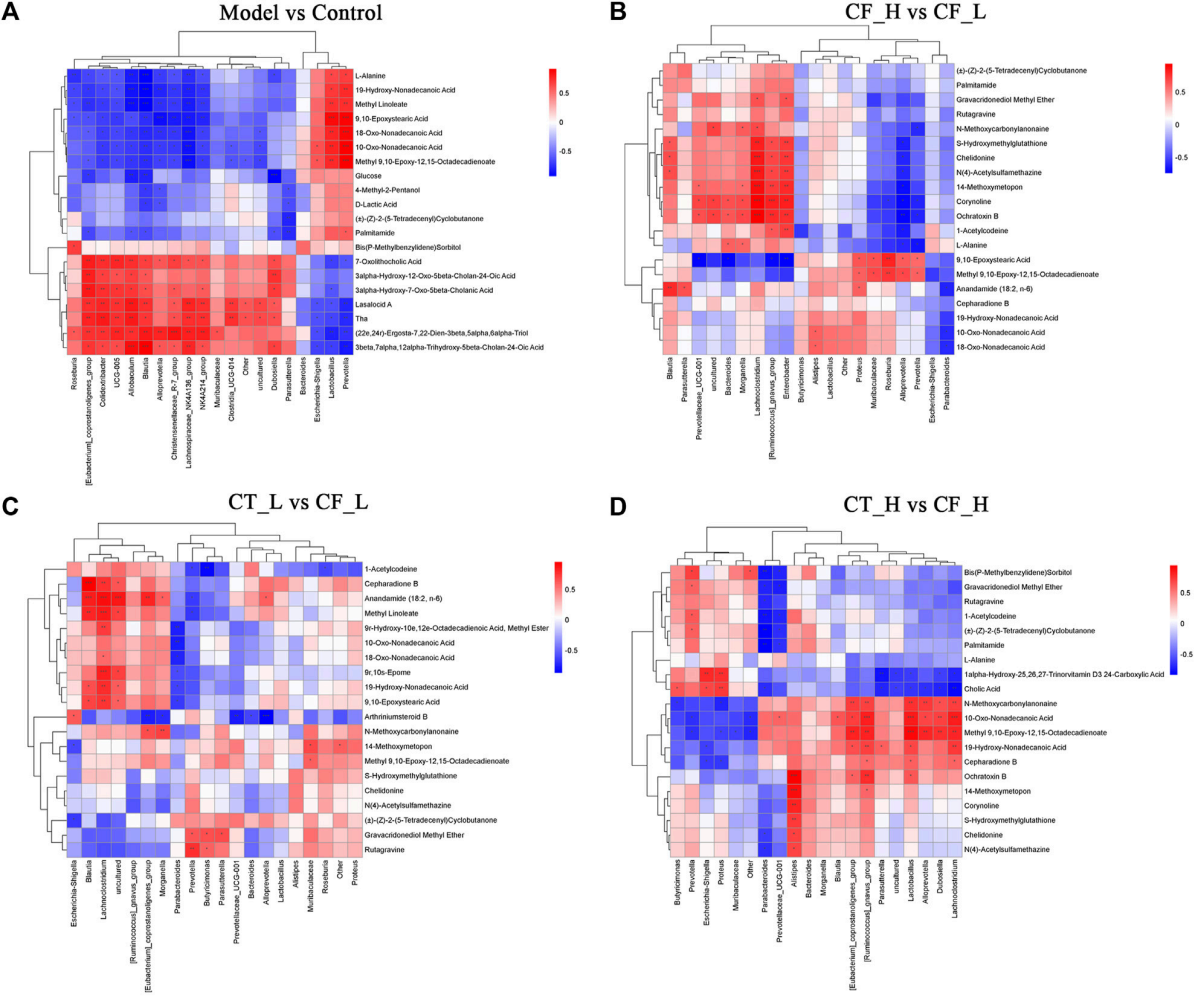
Correlation analysis of fecal metabolites with fecal microbiota genera based on the Spearman algorithm. **(A)** Model vs Control; **(B)** ZYP_H vs Model; **(C)** CT_L vs CF_L; **(D)** CT_H vs CF_H. Red is a positive correlation, blue is a negative correlation, with darker colors indicating a stronger correlation. ***/**/*, *p* < 0.001/*p* < 0.01/*p* < 0.05.

Based on fecal metabolomics and analyses of microbial diversity analyses, differentially expressed metabolites and microorganisms were subjected to Spearman correlation analyses, thereby assessing the relationship between fecal metabolites and genera of fecal microbiota (Figure 11). Fecal metabolites were found to strongly correlate with fecal microbiota. Differences in the therapeutic effects of different doses of CF and different doses of CF paired with TR on cholestasis may be due to differences in the regulation of both fecal metabolites and fecal microbiota.

## 4 Discussion

Most Chinese botanical drugs have multiple effects. In specific applications, how to control the direction of the effects of Chinese herbal medication by adjusting the dosage and selecting the combination is a classic problem in Chinese herbal medicine prescription science. CF and its commonly used drug combination CT are widely used in treating cholestasis (Yu et al., 2022a; Han et al., 2023b). ALT and AST directly reflected the damage degree of hepatocytes, and ALP and γ-GT were specific and significant markers of cholestasis (Han et al., 2019; Berköz et al., 2021); TBIL, DBIL, and TBA are the crucial indices of bile markers (Zhao et al., 2017). The degree of liver tissue damage and inflammation can be visually assessed by pathological histological observation. In the present study, serum biochemical indices and histopathological observations of the liver showed significant therapeutic effects of both CF and CT on cholestasis. On this basis, we investigated the characteristics of the therapeutic effects of CF on cholestasis at different doses (CF_L, CF_H) and in combination with TR (CT_L, CT_H) through the three perspectives of anti-inflammatory effect, hypolipidemic effect, and anti-oxidative stress effect, respectively. Levels of IL-6, TNF-α, IL-10, and IL-1β in liver tissue reflect the degree of hepatic inflammation caused by cholestasis (Wei et al., 2020; Gallucci et al., 2022), while serum levels of TC, TG, LDL-C, and HDL-C reflect lipid metabolism and oil red O staining visualizes lipid accumulation in liver tissue (Sheng et al., 2019). ROS, GSH, SOD, MDA, and NO reflect cytotoxicity and hepatic injury caused by the accumulation of reactive oxygen species (González et al., 2011; Yao et al., 2017). On the dose, the anti-inflammatory, hypolipidemic, and anti-oxidative stress effects of CF showed a significant quantitative dependence, in which the anti-inflammatory and anti-oxidative stress effects were enhanced with increasing dose; on the contrary, the hypolipidemic effects were weakened with increasing dose. Interestingly, after CF on the combined TR, the two have a synergistic effect regarding hypolipidemia and a potential antagonistic effect regarding anti-inflammation.

Gut microorganisms produce a range of metabolites during colonization and reproduction that directly and indirectly affect host metabolic and immune responses (Coker et al., 2022). Metabolomics technology is an important research method to investigate the effect mechanisms of Chinese medicines in treating diseases. To analyze the reasons for the above differences in efficacy, this study first used fecal metabolome sequencing, Model vs Control, CF_H vs CF_L, CT_L vs CF_L, and CT_H vs CF_H were screened for significant differential metabolites, and these metabolites were analyzed for functional enrichment, respectively. Linoleic acid promotes metabolism, regulates the endocrine system, and encourages lipid metabolism, among other functions (Spector and Kim, 2015). It is also a catalyst for cholesterol metabolism, which reduces the levels of cholesterol and lipids in the blood (Ebrahimi-Mameghani et al., 2016). Previous studies have shown that linoleic acid metabolism is closely related to inflammation and oxidative stress (Khazen et al., 2007). When liver injury occurs, linoleic acid is released from phospholipids and further produces inflammatory mediators, and the expression of linoleic acid is a marker of liver injury (Xu et al., 2021). Arachidonic acid plays a crucial role in allergy, inflammation, and other organ functional responses, and arachidonic acid metabolism can reflect, to some extent, the severity of inflammation (Nakaya et al., 2000; Canfora et al., 2015). Tyrosine is a significant substrate for endogenous peroxidases. The presence of tyrosine and its derivative *in vivo* and *in vitro* could ameliorate oxidative damage through ferryl heme reduction (Lu et al., 2014). Additionally, high levels of tyrosine may promote fatty acid synthesis, further promoting liver fat deposition (Jin et al., 2016).

Various previous studies have shown that ecological disturbances in the gut microbiota are closely related to the development of cholestasis (Tang et al., 2023; Yu et al., 2023), and our preliminary study found that the equipotential pairing of CF and TR could have a positive intervention effect on cholestasis by interfering with gut microbial homeostasis and metabolic homeostasis (Yu et al., 2022b). In this study, differential microbial communities were obtained by LEfSe analyses of between-group comparisons of Model vs Control, CF_H vs CF_L, CT_L vs CF_L, and CT_H vs CF_H, respectively. *Prevotella* is a symbiotic bacterium whose relative abundance has been reported to be associated with inflammation (Ning et al., 2020; Wang et al., 2020), with an enhanced ability to induce inflammatory mediators such as IL-6 and TNF-α (Larsen, 2017). *UCG-005* helps promote the production of short-chain fatty acids (SCFAs), which helps alleviate inflammation and lipid metabolism disorders (Li et al., 2022b; Shi et al., 2023). *Lactobacillus* increase fecal lipid excretion and thereby reduce hepatic lipid accumulation by improving the activity of bile salt hydrolytic enzymes and the number of unconjugated bile acids (Janssen et al., 2017). Prediction of KEGG function in differential microorganisms showed that the difference between low-dose and high-dose CF for cholestasis was related to amino acid biosynthesis, whereas the difference between the therapeutic effects of CF and CT for cholestasis was related to 2-Oxocarboxylic acid metabolism and amino acid biosynthesis. These were similar to the results obtained from fecal metabolomics analyses. Additionally, correlation analyses of microorganisms and metabolites showed the existence of a relationship between the relative abundance of microorganisms and the levels of metabolites.

The anti-inflammatory, lipid-lowering, and anti-cholestatic effects of CF have been reported in many papers (Wang et al., 2019; Hu et al., 2022; Lan et al., 2022), and our previous studies have confirmed that the combination of CF and TR in the treatment of cholestasis is associated with regulating the expression of genes related to lipid metabolism (Han et al., 2023a). The effect of the combination of CF and TR in the regulation of disorders of lipid metabolism has also been demonstrated by other scholars in their studies (Zhang et al., 2022; Xu et al., 2023). Amino acid metabolism is closely related to oxidative stress, e.g., tryptophan is a necessary substrate for melatonin synthesis, while melatonin has a significant anti-oxidative stress effect for treating cholestasis (Yu et al., 2018). In future studies, we will continue to investigate the specific mechanisms by which different dosages and combinations of TR alter CF anti-cholestasis by modulating lipid and amino acid metabolism.

## 5 Conclusion

General, in the treatment of cholestasis, the efficacy of low-dose CF was biased towards hypolipidemic, and high-dose CF was biased towards anti-inflammatory effects; furthermore, CF and TR had synergistic effects on hypolipidemic effects and potential antagonistic effects on anti-inflammatory effects. On biological mechanisms, this may be due to differences in the dual effects on gut microbiology and metabolic homeostasis, of which lipid metabolism and amino acid metabolism are critical events. This study elucidates the mechanism of CF in the treatment of cholestasis and provides new ideas for the clinical application of CF in the treatment of cholestasis.

## Data Availability

The data presented in the study are deposited in the https://www.ncbi.nlm.nih.gov/, https://www.cncb.ac.cn/ repository, accession numbers PRJNA1072646; OMIX005790.
